# Tandem Repeats, High Copy Number and Remarkable Diel Expression Rhythm of Form II RuBisCO in *Prorocentrum donghaiense* (Dinophyceae)

**DOI:** 10.1371/journal.pone.0071232

**Published:** 2013-08-19

**Authors:** Xinguo Shi, Huan Zhang, Senjie Lin

**Affiliations:** 1 State Key Laboratory of Marine Environmental Science and College of Ocean and Earth Sciences, Xiamen University, Xiamen, Fujian, China; 2 Department of Marine Sciences, University of Connecticut, Groton, Connecticut, United States of America; 3 College of the Environment and Ecology, Xiamen University, Xiamen, Fujian, China; 4 Department of Environmental Science, Ocean University of China, Qingdao, Shandong, China; University of Texas Southwestern Medical Center, United States of America

## Abstract

Gene structure and expression regulation of form II RuBisCO (*rbcII*) in dinoflagellates are still poorly understood. Here we isolated this gene (*Pdrbc*) and investigated its diel expression pattern in a harmful algal bloom forming dinoflagellate *Prorocentrum donghaiense*. We obtained cDNA sequences with triple tandem repeats of the coding unit (CU); the 5′ region has the sequence of a typical dinoflagellate plastid gene, encoding an N-terminus with two transmembrane regions separated by a plastid transit peptide. The CUs (1,455 bp except 1464 bp in last CU) are connected through a 63 bp spacer. Phylogenetic analysis showed that *rbcII* CUs within species formed monophyletic clusters, indicative of intraspecific gene duplication or purifying evolution. Using quantitative PCR (qPCR) we estimated 117±40 CUs of *Pdrbc* in the *P. donghaiense* genome. Although it is commonly believed that most dinoflagellate genes lack transcriptional regulation, our RT-qPCR analysis on synchronized cultures revealed remarkable diel rhythm of *Pdrbc* expression, showing significant correlations of transcript abundance with the timing of the dark-to-light transition and cell cycle G2M-phase. When the cultures were shifted to continuous light, *Pdrbc* expression remained significantly correlated with the G2M-phase. Under continuous darkness the cell cycle was arrested at the G1 phase, and the rhythm of *Pdrbc* transcription disappeared. Our results suggest that dinoflagellate *rbcII* 1) undergoes duplication or sequence purification within species, 2) is organized in tandem arrays in most species probably to facilitate efficient translation and import of the encoded enzyme, and 3) is regulated transcriptionally in a cell cycle-dependent fashion at least in some dinoflagellates.

## Introduction

Ribulose bisphosphate carboxylase/oxygenase (RuBisCO) is the first major enzyme responsible for photosynthetic fixation of inorganic carbon and is thus critical in sustaining the ecosystem and in carbon biogeochemical cycling. The enzyme catalyzes carboxylation of ribulose-1,5-bisphosphate to yield two molecules of phosphoglycerate in Calvin-Benson-Bassham pathway while it also catalyzes oxygenation of the same substrate to produce glycolate in photorespiration. There are three classes of RuBisCO, forms I, II and III, all catalyzing the primary CO_2_ fixation reaction [1], [2]. Most common in eukaryotic algae is the form I RuBisCO, which contains eight large subunits (RbcL) and eight small subunits (RbcS), both typically encoded in the plastid genome except in land plant and chlorophyte algae in which RbcS is encoded in the nucleus. Form II RuBisCO (RBCII) is mainly present in proteobacteria, and peridinin-containing dinoflagellates are the only group of eukaryotes known to harbor this type of RuBisCO [3]–[6]. RBCII consists only of the large subunit(s). Apparently acquired through horizontal transfer [3], this gene in dinoflagellates is encoded in the nucleus. Form III RuBisCO has only been found in Archaea so far, and seems to be a dimer of the large subunit [7]. In addition, RuBisCO-like proteins, categorized as form IV RuBisCO, have also been found in some bacteria, Archaea and algae [1], [2], [8], but unlike typical RuBisCO they do not catalyze carbon fixation. Recent phylogenetic analyses show that all the four forms of RuBisCO may have evolved from a common ancestor [1].

Dinoflagellates are arguably the largest group of marine eukaryotic phytoplankton aside from diatoms, and one of the most important primary producers in the marine ecosystem. Besides, dinoflagellates have profound impacts on coral reef due to indispensable endosymbiotic association of *Symbiodinium* spp. with corals, and on coastal health and economy as the major contributors of harmful algal blooms (HABs) and algal toxins (for review, see [9]). Understanding the structure and expression regulation of RBCII gene (*rbcII*) in dinoflagellates would provide insights into the trend and mechanism in the evolution of this important enzyme and shed light on how the activity of this enzyme is regulated and in turn contribute to the success of dinoflagellates under the diverse ecological settings.

So far only four dinoflagellate species have been studied regarding *rbcII*, *Gonyaulax polyedra* [3], *Symbiodinium* sp. [4], *Prorocentrum minimum* [5], and *Heterocapsa triquetra* [6]. This gene is arranged as tandem repeats in the genomes of all the species but *H. triquetra*. Triple and quadruple tandem repeats of *rbcII* coding units (CUs) were found respectively in *Symbiodinium* sp. [4] and *Prorocentrum minimum* [5]. The tandem repeats were linked by a short spacer sequence. In *P. minimum*, 148±16 CUs were estimated, which are believed to be organized in 37±4 transcribed quadruple-tandem-repeat units [5]. Overall, information of *rbcII* in dinoflagellates is still very limited.

In this study, we isolated *rbcII* from the HAB forming dinoflagellate *P. donghaiense* (*Pdrbc*). This species (also known as *P. dentatum*) is widely distributed in Chinese, Japanese, Korean and Turkey coasts, and regularly forms HABs in the Chinese coastal waters [10], [11]. We analyzed *Pdrbc* gene structure, quantified its copy number, and investigated the expression pattern under light/dark cycle as well as continuous light or dark conditions. The knowledge of *Pdrbc* expression regulation would be helpful for understanding the regulatory mechanism of photosynthetic growth and bloom formation in this species. As a species phylogenetically close to *P. minimum* [12], the analysis of this gene in *P. donghaiense* also offers an opportunity to examine the evolution of *rbcII* in closely related dinoflagellate species.

## Methods

### Algal culture and sample collection for RuBisCO gene structural analysis

*P. donghaiense* culture was grown in a glass bottle containing 1-L of L1 medium (without silicate) prepared with filtered (0.22 µm), autoclaved seawater at 20±1°C under a 14∶10 h light∶dark cycle with a photon flux of 100 µE m^−2^ s^−1^. The culture was verified to be *P. donghaiense* free of eukaryotic contaminants by microscopic examination and direct sequencing of the PCR product of 18S ribosomal RNA gene amplified with a eukaryotic universal primer set [13] using genomic DNA of the culture (prepared as shown below) as the template. To monitor the growth rate of the culture, cell concentration was measured daily using a Sedgwick-Rafter counting chamber under a microscope.

Samples were collected for nucleic acid extraction. For RNA, about 10^7^ cells were collected in the exponential growth phase by centrifugation at 3000×g at 20°C for 10 min. The cell pellets were resuspended in 1 ml TRIzol Reagent (Invitrogen, Carlsbad, CA) and stored at −80°C for subsequent RNA extraction (usually within a month). For genomic DNA extraction, additional samples were collected as described above and cell pellets were suspended in 400 µl DNA lysis buffer (0.1 M EDTA, pH 8.0, 1% SDS) and stored −20°C for DNA extraction later.

### Culture synchronization and light/dark manipulations to study *Pdrbc* expression pattern

A synchronized *P. donghaiense* culture was produced as follows: a stock culture was grown in a bottle containing 5-L L1 medium and dense cell population flocking in the upper portion of the culture suspension was transferred weekly to a new bottle filled with fresh L1 medium. This procedure was repeated four times in a month and the synchrony of the culture was confirmed by flow cytometry analysis of PI (Propidium Iodide) DNA-stained samples (see below). The synchronized culture was then split to three bottles, which were allowed to acclimate for a day. Next day, after the new cultures were confirmed to be in the exponential growth phase (average specific growth rate, calculated as [ln(N_2_)–ln(N_1_)]/(t_2_–t_1_) where N2 and N1 are cell concentrations at time t2 and t1, respectively, was 0.76 d^−1^), we conducted a 24-h diel sampling. Each of the triplicate cultures was sampled every 2 h, starting two hours before the onset of the dark period. At each time point, 300 ml and 50 ml samples were collected as described above for RNA extraction and flow cytometry assay, respectively. RNA samples were thoroughly suspended in TRIzol and stored at −80°C whereas flow cytometry samples were suspended in 5 ml 1% PFA and stored at 4°C.

To separate the effect of the light/dark cycle from that of the cell cycle on *Pdrbc* expression rhythm, synchronized cultures grown as described above were transferred to continuous illumination or continuous darkness when the light cycle finished, with other conditions remaining unchanged, and diel sampling was conducted as mentioned above for each of the triplicated cultures after they had been placed in the continuous light and continuous dark for 22 h, respectively. For the cultures grown in continuous darkness, care was taken not to expose the cultures to light by covering the culture bottles with multi-layered black cloth and having all the lighting turned off, leaving only a dim red light in the room, and each sampling was carried out as quickly as possible. Samples were stored in TRIzol and kept at −80°C as described above for RNA extraction later.

### DNA and RNA extraction and cDNA synthesis

Four µl of Proteinase K (final concentration 200 µg µl^−1^) was added to the *P. donghaiense* sample preserved in DNA lysis buffer, and the sample was incubated for 3 days at 55°C, with the prolonged incubation intended to maximize cell breakage. DNA extraction was then carried out using a CTAB protocol combined with Zymo DNA Clean & Concentrator kit (Zymo Research Corp., Orange, CA) as previously reported [13].

Total RNA was extracted using TRI-Reagent protocol (Molecular Research Center, Inc., Cincinnati, OH) coupled with Qiagen RNeasy Mini kit (Qiagen), as reported [14]. To eliminate potential genomic DNA contamination, the RNA samples were incubated with RQ1 DNase (Promega) for 30 min at 37°C and further purified using Qiagen RNeasy Mini kit. RNA concentration was measured using a NanoDrop ND-2000 Spectrophotometer (ThermoScientific, Wilmington, DE, USA), while quality assessed using RNA 6000 Nano LabChip Kit in microcapillary electrophoresis (Agilent 2100 Bioanalyzer, Agilent Technologies, Australia). The RNA integrity number (RIN) of the samples (>6.0) was all above the recommended value (5.0). The same amount (200 ng) of the total RNA was used in cDNA synthesis for each sample. For RuBisCO cDNA isolation, GeneRacer oligo-dT (Invitrogen, Carlsbad, CA, USA) and modified random N9 primers ([15], Table 1) were used separately to construct cDNA libraries (named Racer3′-cDNA and N9-cDNA, respectively). For quantitative PCR, oligo-(dT)16 primer was used in the cDNA synthesis.

**Table 1:** Primers used in this study.

Primer name	Sequences (5′–3′)	Application	Source
Rbc5a	CTGTTCGACCGCAACMTCACCGATGG	Dinoflagellate *rbcII* PCR (forward)	[5]
Rbc3a	CCAAGCTTCTCCTTCCAKCCHGGRTADAT	Dinoflagellate *rbcII* PCR (reverse)	[5]
Dino-SL	TCCGTAGCCATTTTGGCTCAAG	Dinoflagellate mRNA 5′ end cDNA synthesis and PCR (forward)	[16]
Pdrbc5b	GCAACTTCCCGAAGCAGTTCCTGCA	*Pdrbc* 3′ end isolation	This study
Pdrbc3b	GTACTTGCCACGCGCGATCATCTCA	*Pdrbc* 5′ end isolation	This study
GeneRacer oligo-dT	GCTGTCAACGATACGCTACGTAACGGCATGACAGTG(T)24	cDNA library construct	Invitrogen
GeneRacer3	GCTGTCAACGATACGCTACGTAACG	cDNA library construct	Invitrogen
Regular oligo-dT	(T)_16_V (V = A,G,C)	cDNA library construct	[5]
Modified random N9	GAGACTATGCGCCTTGCCAGCCCGCTCAGTAATACGACTCACTATAGGGAGNNNNNNNNN	cDNA library construct	[15]
Rbc5F	CTGGACYAGTCGAGCCGYTAYGCKGA	*Pdrbc* PCR (forward)	This study
Rbc3R	CTCCTTSTTCTGCCARTTGAAGCTRGC	*Pdrbc* PCR (reverse)	This study
Rbc5H	GGCTGGAAGGAGAAGCTCGGCTACAC	*Pdrbc* PCR (forward)	This study
Rbc3H	CGGTCGGGTAYGCAATyTTCATCTC	*Pdrbc* PCR (reverse)	This study
Rbc-QF	GCGAAGACCCATGAGGAGATCAAG	*Pdrbc* qPCR (forward)	This study
Rbc-QR	TCAACTCAACTCCTTCTTCTGCCAAT	*Pdrbc* qPCR (reverse)	This study
Pdong-cal-QF	AGTTCAAGGAGGCGTTCTCTTTGTTC	*P. donghaiense calmodulin* qPCR (forward)	This study
Pdong-cal-QR	CCATCAAGGACAAGAACTCGGGAAAG	*P. donghaiense calmodulin* qPCR (reverse)	This study
Pdong-gapdh-QF	GTGTTCCTYACCGACGAGAAGATC	*P. donghaiense gapdh* qPCR (forward)	This study
Pdong-gapdh-QR3	CGCARTTCATGTCAGTCTTGTAGG	*P. donghaiense gapdh* qPCR (reverse)	This study

### Isolation of *Pdrbc* cDNAs and analysis of tandem repeats

Primers used in this study, designed previously or in this study, are shown in Table 1. Primer set Rbc5a-Rbc3a was used to PCR amplify a cDNA fragment of *Pdrbc*. The amplicon was purified and sequenced as reported [13]. *Pdrbc* Specific primers Pdrbc3b and Pdrbc5b were designed according to the sequence and used in rapid amplification of cDNA ends (RACE) to isolate the 5′ and 3′ ends of *Pdrbc* cDNA. The dinoflagellate cDNA 5′-end common sequence, spliced leader or DinoSL [16], paired with dinoflagellate *rbcII* specific primer Pdrbc3b, was used in PCR with N9-cDNA library as the template using TAKARA ExTaq Polymerase (TAKARA Biotechnology, Dalian, Liaoning, China), to isolate the 5′-end *Pdrbc* sequence. PCR was performed using a touch-down protocol (95°C 25 s, 68°C 75 s for five cycles, followed by 95°C 15 s, 58°C 30 s, 72°C 45 s for 30 cycles, and a final step of 72°C for 5 min). For the 3′-end sequence isolation, primer Pdrbc5b was paired with Racer3 in PCR with GeneRacer cDNA library as the template. The amplicons of both the 5′- and 3′-ends of cDNA were purified, cloned, and sequenced.

Primer set Rbc5F-Rbc3R was designed to amplify a complete CU of *Pdrbc* based on the sequences obtained above and reported previously for other dinoflagellates. PCR was carried out using the touch-down protocol mentioned above and the PCR product was purified, cloned, and sequenced. Primer set Rbc5H-Rbc3H was used in PCR to obtain the CU spacer sequence. The primer was designed according to the C-terminal and N-terminal sequences of the CU obtained above. In order to detect potential polymorphic sites, the PCR products were sequenced both directly and after cloning.

To amplify full-length cDNA from mature mRNA, several primers were designed in the 5′-UTR and 3′-UTR regions of *Pdrbc*. These primer sets were paired with one another or with DinoSL/Racer3 in the PCR using TAKARA-LA Taq Polymerase. In order to enhance the chance of success in amplifying long transcripts that contained multiple CUs, long elongation time (8 min) was used in PCR as reported [5].

### Phylogenetic analysis to examine the evolutionary trajectory of dinoflagellate RuBisCO gene duplication

Sequences obtained were searched against GenBank non-redundant nucleotide and protein databases using Basic Local Alignment Search Tool (BLAST). Top hits were collected and aligned with our sequences using ClustalX [17]. Phylogenetic analyses were performed using Neighbor-joining (NJ) [18] and Bayesian methods [19]. The branching patterns of the gene copies within and between species were examined to determine whether RuBisCO gene duplication occurred before or after speciation.

### Prediction of structural features of *Pdrbc*-encoded protein (PdRBC)

To look for potential signal peptide sequences at the N-terminus, web software Tmpred (http://www.ch.embnet.org/software/TMPRED_form.html) was used to predict the transmembrane regions in the first 124 aa. For comparison, the N-terminal of other reported dinoflagellate RuBisCO sequences (AAW79358 and AAG37859) were included in this analysis. Chlorop V1.1 program (http://www.cbs.dtu.dk/services/ChloroP/) [20] was used to find chloroplast transit peptide (cTP) and their cleavage site. Finally, NetPicoRNA V1.0 program (http://www.cbs.dtu.dk/services/NetPicoRNA/) was used to predict potential posttranslational cleavage sites.

### Quantification of *Pdrbc* copy number using qPCR

The quantitative PCR reactions were performed with genomic DNA as templates using iQTM SYBR® Green Supermix in 96-well plates on a CFX96 Real-time PCR System (BioRad, USA). Each reaction was carried out in a total volume of 12 µl containing 250 nM of each primer, 5 µl DNA (corresponding to 1, 5, and 10 ng DNA separately), and 6 µl 2×SYBR® Green Supermix. To prepare a standard curve, the complete CU of a cloned *Pdrbc* cDNA was PCR amplified; original un-linearized plasmid clones were avoided due to its tendency to overestimate gene copy numbers [21]. The amplicon was purified, quantified using NanoDrop, and then serially diluted by 10-fold to obtain a dilution series of 10^2^–10^7^ gene copies per 5 µl. The standard series and the *P. donghaiense* genomic DNA samples were run on the same PCR plates using the thermo cycle program as reported [5]. All reactions were carried out in three technical replicates. Data was analyzed with CFX software (Bio-Rad, Hercules, CA).

### Gene expression analysis using reverse transcription quantitative PCR (RT-qPCR)

 The expression level of *Pdrbc* was detected using RT-qPCR as described above using cDNAs prepared from the diel RNA samples. The standard used was the same as described above for gene copy quantification. For use as reference genes with which to normalize the expression of *Pdrbc*, we tested five genes including calmodulin (*calm*), glyceraldehyde 3-phosphate dehydrogenase (*gapdh*), *actin*, *α-tubulin* and mitochondrial cytochrome *b* (*cob*) for the diel samples. Using GenNorm (http://ikmbio.csie.ncku.edu.tw/GN/), we analyzed relative expression stability for these genes, and found that the expression of *gapdh* and *calm* was more stable than the other three, although none of them showed a constant expression level. *Gapdh* has been shown as a relatively good reference gene (i.e. expression level relatively stable) in other organisms including dinoflagellates ([22] and the refs therein), while calm is one of the highly expressed genes in dinoflagellates under various growth conditions [9]. As a result, *gapdh* and *calm* were selected. Their standards for RT-qPCR were prepared by PCR in the same way as *Pdrbc* standard. Each cDNA template was analyzed in triplicate. *Pdrbc* transcript abundances resulting from RT-qPCR were normalized to the transcript abundances of the two reference genes (*calm* and *gapdh*) as well as the quantity of total RNA (QTR) equivalent to the amount of cDNA used in each reaction.

### Flow cytometric analysis of the cell cycle

Samples fixed in 1% PFA were centrifuged (2000×g, 10 min, 4°C) to pellet the cells, and washed with 1×PBS (phosphate buffered saline, PH = 8.0). The pellet was resuspended in 5 ml ice cold absolute methanol (to extract pigments that could interfere with DNA fluorescence measurement and permeabilize the cells for easier entry of DNA-staining dyes) and stored at 4°C for 12 h. The cells were again pelleted by centrifugation (2000×g, 10 min, 4°C) and resuspended in 1×PBS. Then the pellet was resuspended with 0.5 ml propidium iodide (Sigma, St. Louis, MO) staining solution containing 100 mM Tris, pH 7.4, 150 mm NaCl, 1 mM CaCl_2_,0.1% NonidetP-40, 0.1 mg ml^−1^ RNase, and incubated in the dark for 1 hour at 37°C to digest RNA and stain DNA. DNA analysis of the PI stained cells were carried out on an Epics XL flow cytometer (Beckman Coulter, Miami, FL) with 488 nm and 635 nm laser excitation and emission wavelength, which collected signals from 20,000 randomly encountered cells for each sample. Based on the flow cytometric data, the cell cycle profile in each sample was analyzed using MultiCycle software.

### Correlation analysis of *Pdrbc* expression level with potential regulating factors

Linear regression was used to seek correlations between normalized *Pdrbc* transcript abundance and time distance from the dark-to-light transition as well as the fraction of cells in G1, S, and G2M phases of the cell cycle. Fisher's exact tests were conducted for analyzing qualitative differences. A *p*-value of 0.05 was considered the threshold of statistical significance. The statistical analysis was performed using R (2.14.0) software.

## Results

### *Pdrbc* cDNA structure

**Figure 1:**
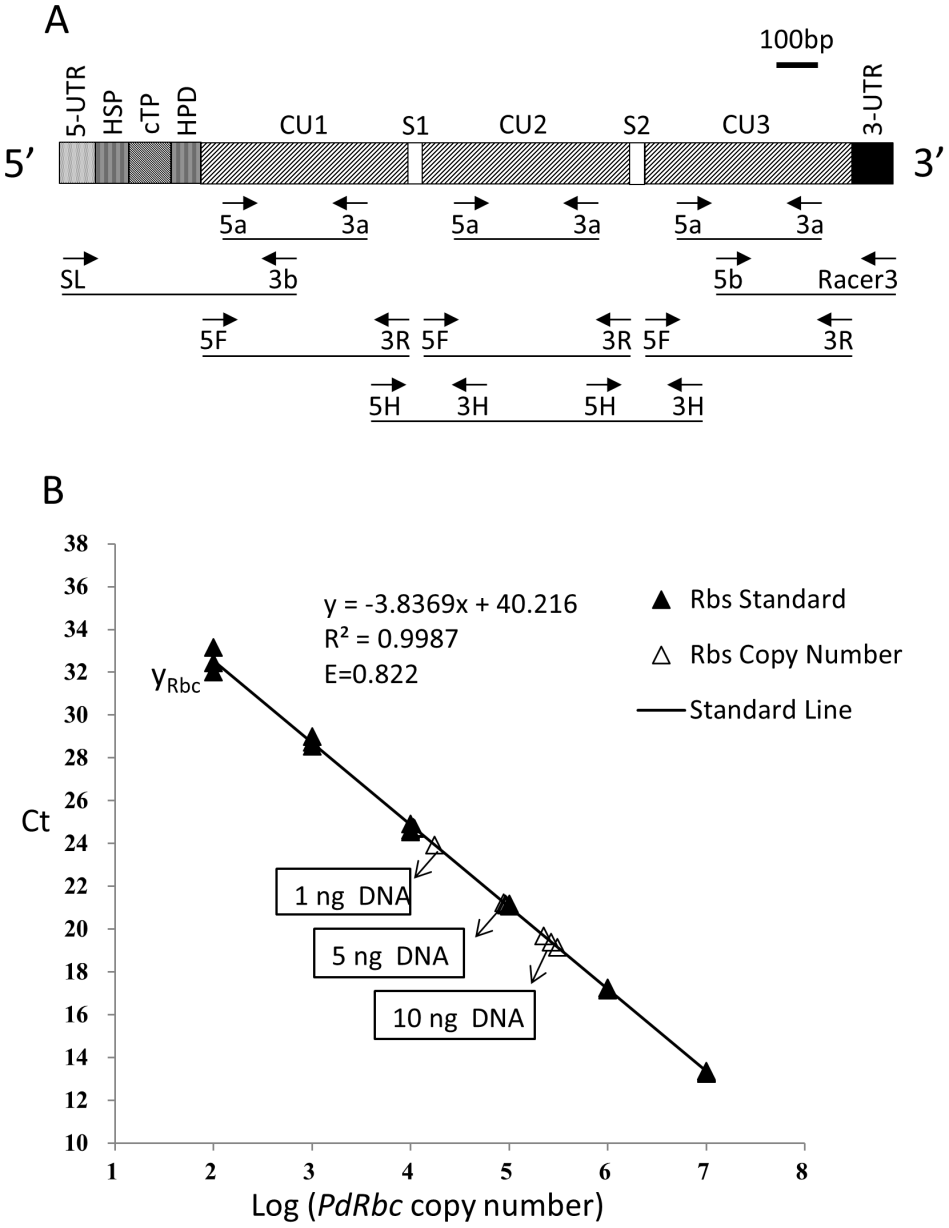
*P. donghaiense* form II RuBisCO gene (*Pdrbc*) structure and copy number in the genome. A) Gene structure (horizontal bars) inferred from isolated cDNA sequences using various primer sets (arrows with labels below the bars). 5-UTR, 5′-untranslated region; HSP, hydrophobic signal peptide; cTP, chloroplast transit peptide; HPD, hydrophilic domain; CU, coding unit; S, spacer sequence; 3-UTR, 3′-untranslated region; The primer sequences are shown in Table 1. B) Estimation of *Pdrbc* copy number in *P. donghaiense* genome using qPCR. Standard curve (solid triangle and line) was constructed using decadal dilution series of purified *Pdrbc* cDNA, from 10^7^ to 10^2^ copies, each in triplicate. Three different amounts of *P. donghaiense* genomic DNA (1 ng, 5 ng and 10 ng), each in triplicate, were used as unknown samples, which gave estimate of 117±40 copies of Pdrbc in one cell.

Using the primer set DinoSL-Pdrbc3b (Figure 1A, Table 1), a 5′-end cDNA fragment (1,069 bp) was amplified from the N9-cDNA library. BLASTX of the sequence against GenBank database showed that its 3′ region (606 bp) was 91% identical to that of *P. minimum rbcII* (*PmrbcII*; AAO13049) at the amino acid (aa) level, but the upstream 463 bp did not hit any reported sequence. The result of NCBI ORF Finder (http://www.ncbi.nlm.nih.gov/gorf/gorf.html) analysis suggested that the coding region started at position nt 109 where methionine codon (ATG) was located and the ORF continues till the end of the 1,069 bp fragment (predicting 320 amino acid residues or aa). The first 108 bp was the 5′-untranslated region (5′-UTR) and contained DinoSL at the 5′ end.

PCR using primer set Rbc5a-Rbc3a (Figure 1A, Table 1) with Racer3′ cDNA library as the template yielded a 1,136 bp cDNA. The sequence obtained from this product was 96% identical (at aa level) to *PmrbcII*. With 3′-RACE, eight 3′-end cDNA clones of *Pdrbc* were obtained, yielding a 167aa partial coding region, a 3′-end noncoding region and a poly(A) tail. Their deduced aa sequence shared 96–97% identity with *PmrbcII* 3′-end sequence (AY169223).

The complete CU of *Pdrbc* was amplified using primer set Rbc5F-Rbc3R. The PCR product was 1,455 bp in length encoding a 485 aa protein (Figure 1A). Eighteen clones of this amplicon along with 23 partial clone sequences were used to detect polymorphic positions in the CU. The CU harbors 142 synonymous and 4 nonsynonymous nucleotide substitutions.

To detect tandem repeats of the CU, PCR was carried out using the oligo-dT synthesized cDNA library with outward primer set Rbc5H-Rbc3H (Figure 1A, Table 1). Two clear bands were obtained, 360 bp and 1,940 bp, respectively. As shown in Figure 1A, the shorter fragment contained a spacer sequence in the middle and two partial CUs at each side, whereas the longer fragment contained one complete CU flanked by two partial CUs. These results indicated that *Pdrbc* transcripts contain at least three CUs. In the attempt to amplify all the CUs in a TU using DinoSL, GeneRacer3 and the primers designed in the 5′- and 3′- UTRs, no detectable amplicon was obtained for any combination of these primer sets.

### *Pdrbc* gene copy number in *P. donghaiense* genome

As shown in Figure 1B, the standard curve for *Pdrbc* qPCR was highly linear (R^2^ = 0.999, efficiency 0.822). Based on the standard curve, qPCR for templates of 1 ng, 5 ng and 10 ng *P. donghaiense* genomic DNA gave estimates of 1.30×10^4^±3.87×10^3^, 9.10×10^4^±3.22×10^3^, 2.66×10^5^±4.23×10^4^ copies (CUs) of *Pdrbc* respectively. These values yielded 1.93×10^4^±6.6×10^3^ *Pdrbc* copies per 1 ng genomic DNA on average (Figure 1B). Based on the spectrophotometrically measured extracted DNA content in *P. donghaiense* (6.04±0.42 pg cell^−1^), there are 117±40 CUs of *Pdrbc* in one cell.

### Phylogenetic relationships of dinoflagellate *rbcII* copies

**Figure 2:**
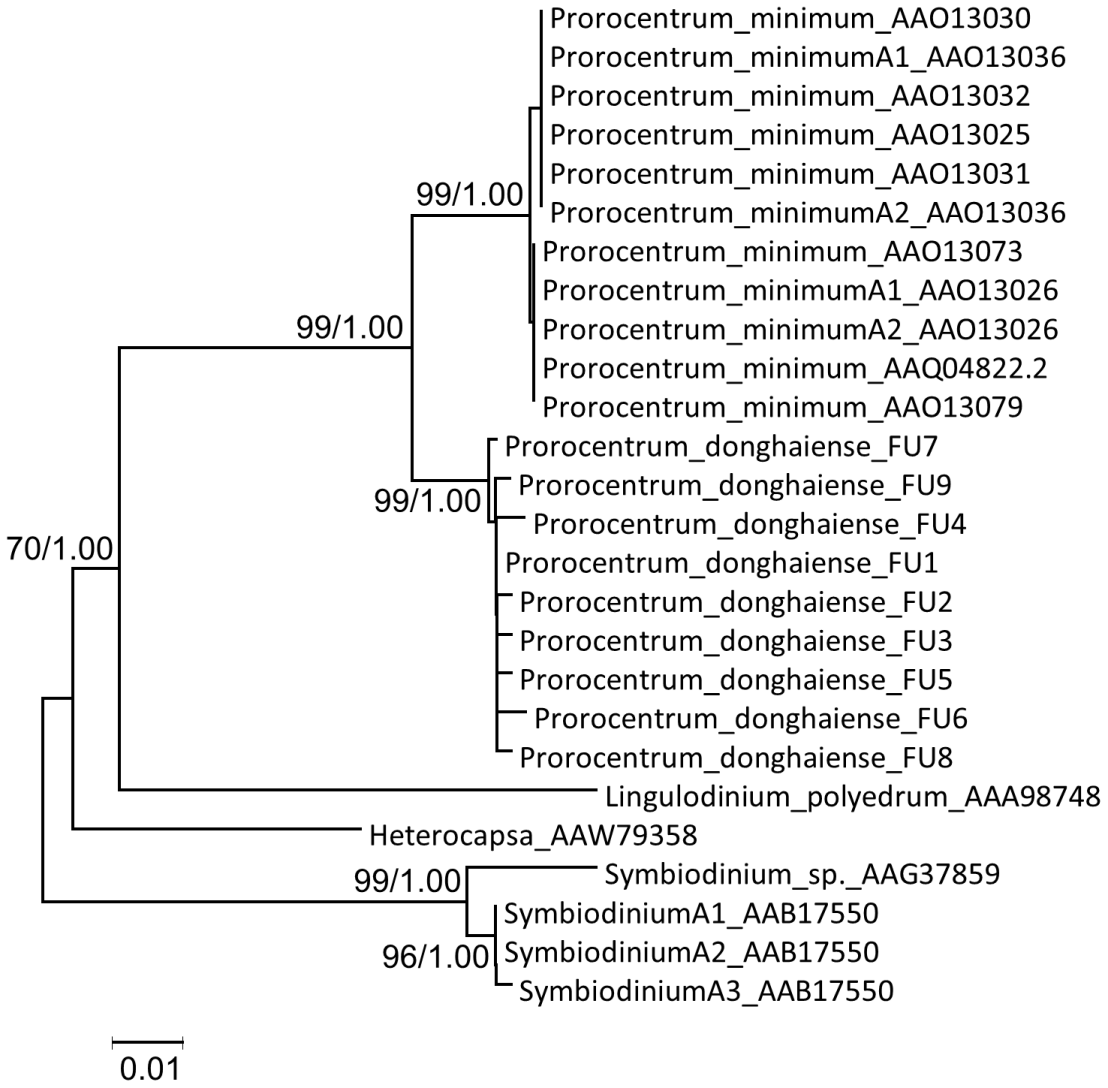
Phylogenetic tree of dinoflagellate form II RuBisCO based on full CU sequences. Tree topology shown is from Neighbor-Joining (NJ) analysis; Bayesian analysis (BE) gave similar tree topology. Support of nodes >70% in NJ bootstrap values (left) and >0.70 in Bayesian posterior probability (right) are both shown.

Phylogenetic analysis of the coding region showed that *Pdrbc* sequences were tightly clustered with those of *P. minimum* (Figure 2). The *Prorocentrum* cluster was allied with *Lingulodinium* and *Heterocapsa*, with that of *Symbiodinium* branched at the base of the few dinoflagellate species studied so far. All the CUs within a species were clustered together forming a monophyletic group.

### Features of PdRBC N-terminus

Signal peptide prediction for PdRBC N-terminus using software SignalP (www.cbs.dtu.dk/services/SignalP/) yielded no significant signal peptide. However, when using Tmpred (http://www.ch.embnet.org/software/TMPRED_form.html), two distinct transmembrane regions separated by an S/T rich region were detected (Figure S3). When we analyzed the aa sequences for the N-terminus of RBCII of the other dinoflagellates (AAW79358 and AAG37859), similar regions were also found. The serine- and threonine-rich feature in this peptide suggested that PdRBC N-terminal tend to form cTPs. Furthermore, ChloroP V1.1 program predicted that the N-terminus contains a cTP with a significant statistical score (0.533); in the analysis amino acid residues 25 to 62 received high scores, suggesting that this region is an important part of the cTP [20].

Downstream of the putative cTP cleavage site is a hydrophobic domain (14 aa). This structure also exists in the N-terminus of RuBisCO in *Symbiodinium* sp. [23] and *H. triquetra* [6]. The hydrophobic domain in RuBisCO is thought to be a mechanistic requirement dictated by the triple-membrane architecture of the plastids [23]. This peptide has been reported to be a stop-transfer membrane domain for translocation in Golgi-derived vesicles, which are essential for transporting RuBisCO protein to the front of the innermost chloroplast membrane [24]. The rest of the N-terminus of PdRBC shows 85–91% aa similarity to RBCII in other dinoflagellates.

### Cell cycle profiles of light/dark (LD), continuous light (LL), and continuous dark (DD) *P. donghaiense* samples

**Figure 3:**
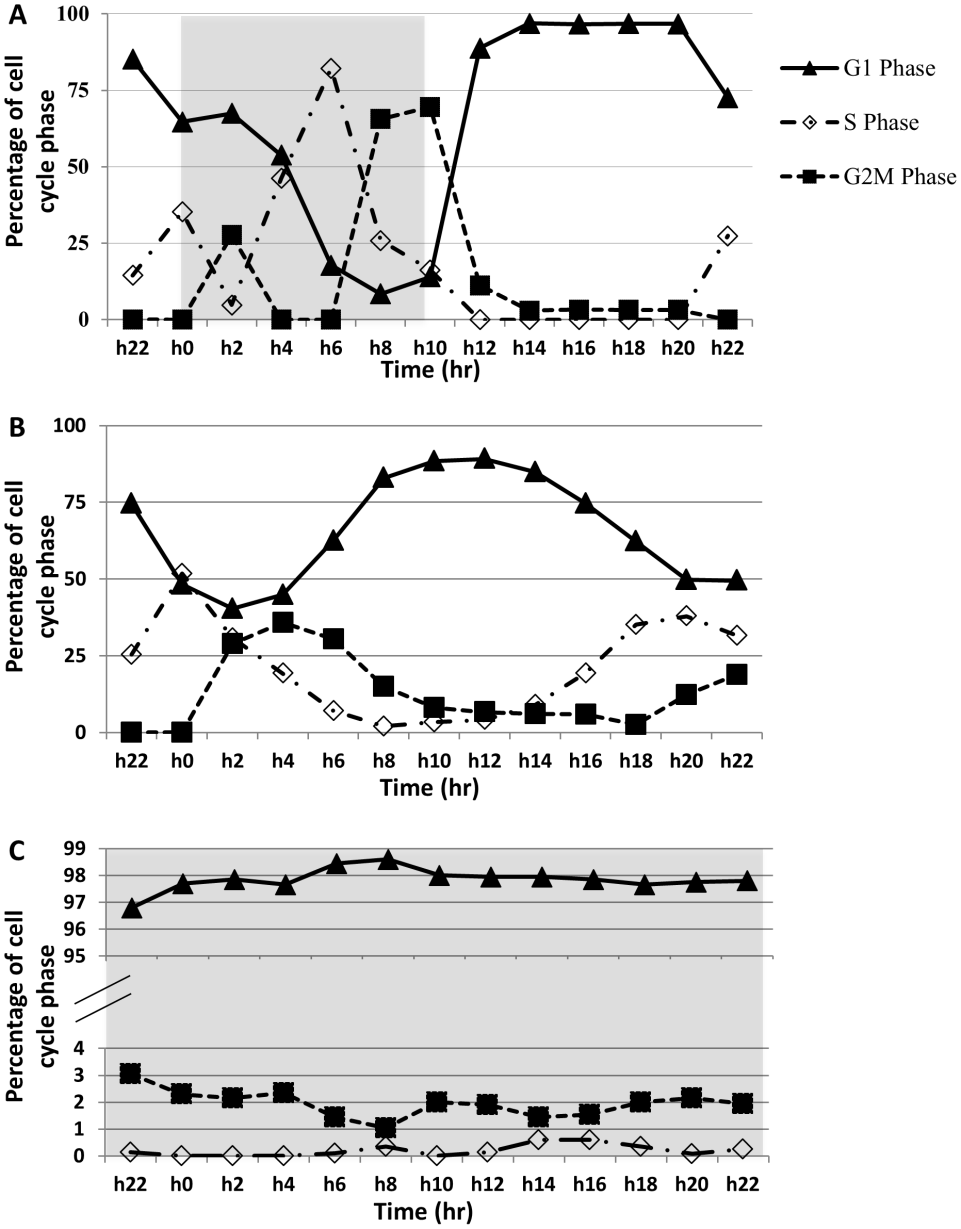
Cell cycle profiles over a 24-h sampling period in the 14∶10 light∶dark cycle (A), continuous light (B) and continuous dark (C) cultures. Grey shading indicates dark period. The progression of the cell cycle can be observed from the time-sequential succession of the peaks of the G1, S, and G2M phase fractions (e.g. A and B), the lack of which is indication of no cell cycle progression (C).

In the 14∶10 LD samples analyzed, active cell division occurred between the later part of the dark period and early part of the light period (Figure S1). With a growth rate of 0.76 d^−1^ (1.13 doubling d^−1^), there were two cell division bursts, one between h4 and h6 and the other from h10 to h14 (Figure S1). The result of the cell cycle analysis (Figure 3A) showed that two hours after light off (h2), the culture started to enter the S phase, with a peak (82.2%) of S-phase fraction (percent cells in the S phase) observed at h6 (4 hours before light turn-on) when G2M-phase fraction started to rise. The G2M-phase peak (69.6%) occurred at the dark/light transition (h10), followed by the increase in the G1-phase fraction. To our knowledge, this was the most-synchronized dinoflagellate culture ever reported.

In the LL samples, flow cytometric analysis of the 24-h samples showed two cycles (Figure 3B), with two peaks of the S-phase, one at h0 and the other at h20, two peaks of G2M-phase that occurred at h4 and somewhere between h22 and h0 respectively, and two G1-phase peaks appearing at h12 and h22, respectively. In all the three phases, the second peak was more pronounced than the first. These peaks were smaller than those in the LD samples. The DD samples were continuously dominated by G1 phase (>96.8%), showing little progression of the cell cycle to the S and the G2M phases (Figure 3C).

### Transcript levels of *Pdrbc* in the 24-h sampling period

**Figure 4:**
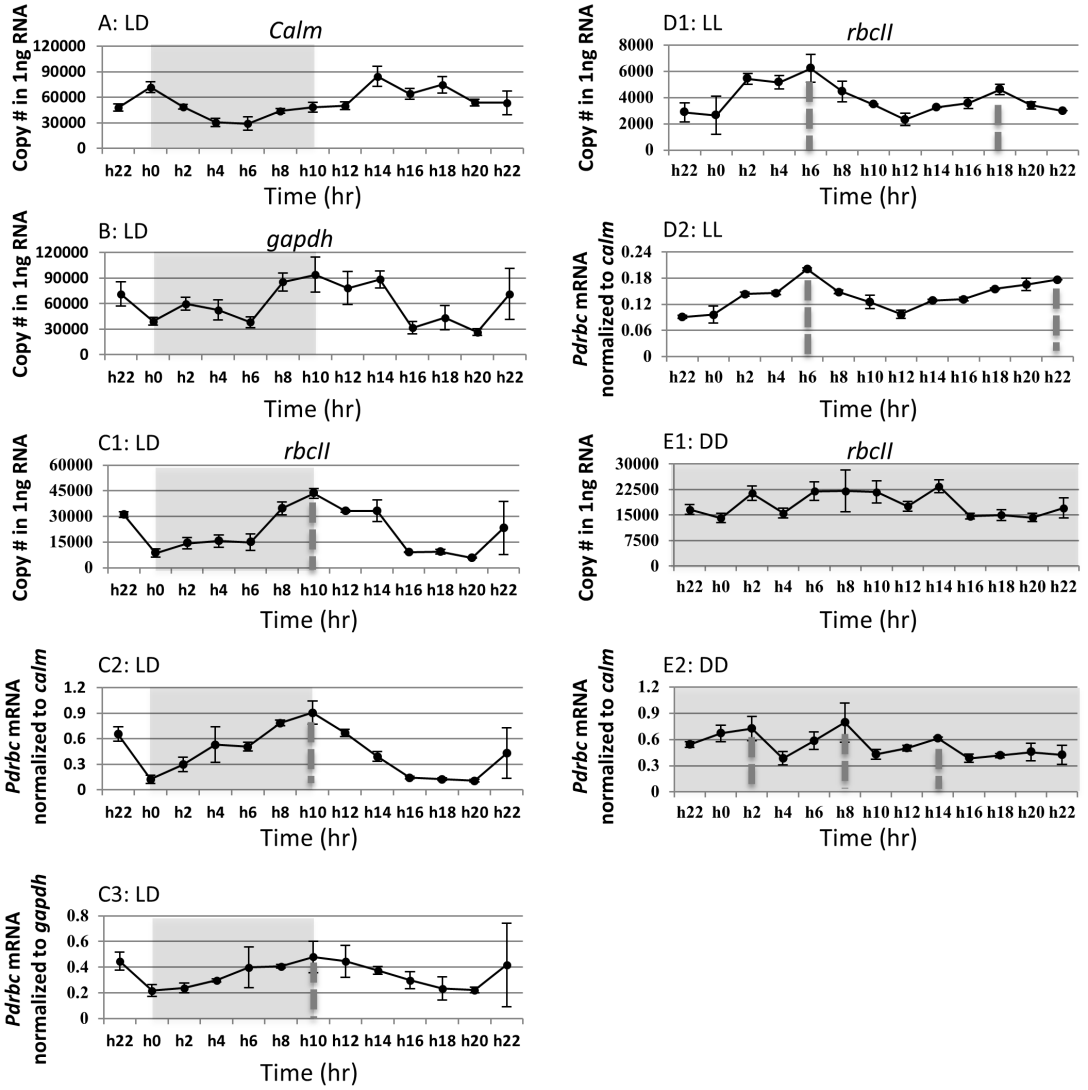
Gene transcription dynamics of reference genes (A–B) and *Pdrbc* normalized to total RNA (C1, D1, E1) and to the reference genes (C2, C3, D2, E2). LD: under light/dark cycle. LL: under continuous light. DD: under continuous darkness. Sampling of the LL and DD cultures started after the cultures had been placed under LL and DD conditions for 22 h, respectively. Hatched bars denote dark cycle. Dotted lines in vertical denote the peak expression time point. Error bars indicate ± standard deviation.

For use in normalizing *Pdrbc* expression, *calm* and *gapdh* (GenBank accession numbers KF142733 and KF142734) were isolated from *P. donghaiense* and specific primers were designed for RT-qPCR. In the LD samples, the expression of *calm* was 2.9-fold lower in the dark than the rest of the diel cycle (Figure 4A). The expression of *gapdh* was highest at late dark period and early light period, with a 3.5-fold variation throughout the diel cycle (Figure 4B). Despite the somewhat different expression dynamics that made these not ideal reference genes, *Pdrbc* expression normalized to them showed similar expression profiles (Figure 4C2,C3), which resembled the profile when *Pdrbc* was normalized to QTR (Figure 4C1); in all case, the normalized *Pdrbc* transcript abundance exhibited a clear diel rhythm (Figure 4C1–C3). At the beginning of dark period, *Pdrbc* transcript abundance was in a lower level. Then it increased steadily from the middle of dark cycle (h6) to reach a maximum at the dark/light transition (h10). The transcript level declined thereafter until the pre-dark period (h20). Throughout the diel cycle, the amplitude of *Pdrbc* transcript dynamics was ∼7.5 fold. In order to test if this result was reproducible, another set of (duplicated) diel samples was collected and *Pdrbc* transcript abundances measured again; the same expression pattern was observed.

In LL samples, there appeared to be two peaks of *Pdrbc* transcripts although the amplitude was smaller than that in the LD culture, with a 2.7-fold variation for *Pdrbc* transcript abundance when normalized to QTR (Figure 4D1) and 2.2-fold when normalized to *calm* (Figure 4D2). In the DD samples, no rhythm of *Pdrbc* expression was apparent when normalized to total RNA (Figure 4E1) and multiple weak peaks were detected when normalized to *calm* (Figure 4E2).

### Correlation between normalized *Pdrbc* mRNA abundance and cell cycle status as well as light/dark cycle

**Figure 5:**
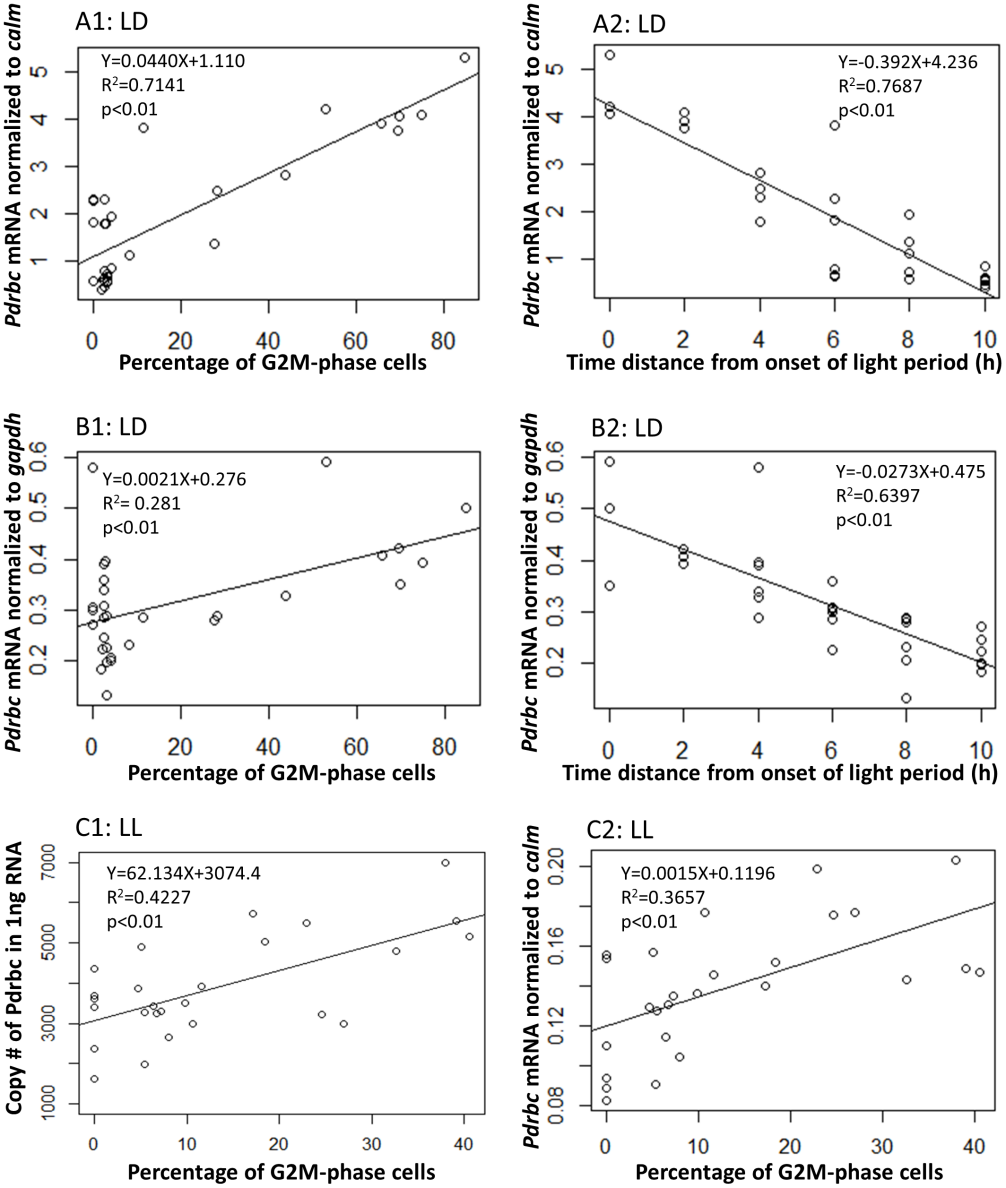
Correlation between *Pdrbc* mRNA abundance and percentage of G2M-phase cells as well as time distance from onset of the light period. A1–A2: correlation between *calm-*normalized *Pdrbc* mRNA abundance and percentage of G2M-phase cells (A1) and time distance (A2) under light/dark cycle. B1–B2: correlation between *gapdh-*normalized *Pdrbc* mRNA abundance and percentage of G2M-phase cells (B1) and time distance (B2) under light/dark cycle. C1–C2: correlation between QTR- or *calm*-normalized *Pdrbc* mRNA abundance and percentage of G2M-phase cells under continuous light. Time distance, regardless before or after onset of the light period, is taken as positive values. As the culture was grown under a 14 h∶10 h light∶dark regime, time points over 10 hours backward from the onset of the light period would be in the light period again, we limited the correlation analysis to data within 10 hours from the onset of the light period. LD: under light/dark cycle. LL: under continuous light. DD: under continuous dark.

For the LD samples, a linear correlation between *Pdrbc* mRNA abundance and G2M-phase fraction was found (Figure 5A,C). The correlation was statistically significant regardless of whether *Pdrbc* mRNA abundance was normalized to *calm* (R^2^ = 0.7141, p<0.01), *gapdh* (R^2^ = 0.281, p<0.01) or QTR (R^2^ = 0.1562, p<0.05; Figure S2). Meanwhile, a significant correlation was also found between *Pdrbc* mRNA abundance and the time distance (taken as positive values regardless it was before or after) from the dark/light transition (Figure 5B,D). The correlation was strong whether *Pdrbc* mRNA abundance was normalized to *calm* (R^2^ = 0.7687, p<0.01), *gapdh* (R^2^ = 0.6397, p<0.01) or QTR (R^2^ = 0.4878, p<0.01; Figure S2).

For the LL samples, a linear correlation between *Pdrbc* transcript abundance and G2M-phase fraction was also found both when *Pdrbc* expression was normalized to *calm* (R^2^ = 0.3657, p<0.01; Figure 5C1) and QTR (R^2^ = 0.4227, p<0.01; Figure 5C2), respectively. However, under the DD condition, no correlation could be detected between *Pdrbc* mRNA abundance and G2M-phase fraction.

## Discussion

Given the importance of dinoflagellates in the marine ecosystem and the essential role of RuBisCO in photosynthetic carbon fixation, the effort to study *rbcII* in dinoflagellates has been disproportionately limited. Only four species have been reported previously [3]–[6]. These studies revealed that *rbcII* in dinoflagellates is likely originated from anaerobic photosynthetic bacteria through horizontal gene transfer; it is nucleus encoded and organized as 3–4 tandem repeats in some species; the enzyme has a significantly lower affinity for CO_2_ and is less stable than form I RuBisCO [4], [25]. In *L. polyedrum*, *rbcII* is reported to be regulated post-translationally (through changes in intracellular localization) [26]. Little information is available on the transcriptional dynamics of dinoflagellate *rbcII*. Our study reported here represents the fifth species of dinoflagellates that have ever been studied in terms of *rbcII* and provides novel information about the structure, evolution, expression pattern, and some clues about regulatory mechanism of this gene.

### High copy number, transcribed unit (TU) organization, and evolution of *Pdrbc*

High copy numbers and tandem-repeat organizations of the gene copies have been reported for genes in dinoflagellates [27], such as genes coding for PCNA [28], luciferin-binding protein [29], small RNAs [16], [30] as well as RuBisCO [4], [5]. A previous study indicated that *P. minimum* genome contains 148 *rbcII* CUs [5]. Comparably, we estimated that *P. donghaiense* genome harbors ∼117±40 *rbcII* CUs. Correspondingly, the genome size of *P. donghaiense* (6.04±0.42 pg DNA cell^−1^ as we measured in this study) is only slightly smaller than that of *P. minimum* (6.9 pg DNA cell^−1^ [31]).

The success of isolating cDNA of *Pdrbc* using DinoSL as the forward primer in PCR verifies the presence of this spliced leader at the 5′ end of *Pdrbc* mRNA, consistent to the postulation that the plastid-originated gene transferred horizontally to the nucleus has adapted to the nuclear environment by acquiring the trait of spliced leader *trans*-splicing [16]. After DinoSL and the 5′-UTR, the rest of the full-length mRNA is composed of a typical dinoflagellate plastid sequence, encoding an N-terminus with two transmembrane regions separated by a plastid transit peptide, then a 120-nt tract that codes for a 40aa hydrophilic domain (Figure S3), and likely followed by the three tandem-arrayed CUs (Figure 1A). The spacer peptide (21aa) that links the neighboring CUs shares 90.5% identities with its counterpart in PmRBC [5].

In contrast to the single-CU *rbcII* transcripts reported for *H. triquetra* [6], our numerous PCR reactions with DinoSL and oligodT as primers never yielded single-CU amplicons. This indicates that all *Pdrbc* CUs are expressed as polyprotein mRNA. Yet also different from the four-CU tandem repeats identified in *Pmrbc* [5], we have only identified three-CU tandem repeats in *Pdrbc*. Even though the long extension time used in PCR supposedly favors long amplicons, none of the many combinations of the outward primers as well as the DinoSL-oligodT pair produced amplicons that contained more than 3 CUs. This result does not completely exclude the possibility that *Pdrbc* mRNA contains four or more CUs but we missed it because it is too long, or contains complex secondary structures for us to amplify it efficiently in PCR. Other analyses such as Northern blotting may be a better way to provide the ultimate proof of the size of the full-length *Pdrbc* mRNA. However, based on current data it is more likely that *Pdrbc* is organized and transcribed as triple-CU tandem repeats because the third CU we retrieved share the same feature as the last (fourth) CU in *Pmrbc*: the 9 additional nt at the 3′ end, which code for Leucine, Serine and a stop codon. In accordance, the detected 117 CUs would perfectly fit in 39 triple-CU TUs. The contrasting architecture of *rbcII* between the two closely related species indicates remarkable divergence in *rbcII* organization that accompanied dinoflagellate speciation.

Interestingly, the triple CU-tandem repeat *rbcII* structure is shared by *Symbiodinium* sp. [4], the species that is not phylogenetically close to the genus of *Prorocentrum* [12], [32]. The tandem repeats of *Pdrbc* start with C(T)TG, similar to its counterparts in other dinoflagellates in which *rbcII* is organized in tandem repeats [3]–[5]. However, in *H. triquetra*, no tandem repeat was found in the cDNA that even contained tansit peptide sequence at the 5′end and polyA tail at the 3′end [6]. These species represent different lineages of dinoflagellates, Prorocentrales (*Prorocentrum* spp.), Gonyaulacales (*L. poledrum*), Suessiales (*Symbiodinium* sp.), and Peridiniales (*H. triquetra*). The differential organizations of these copies between species indicate that the *rbcII* architecture does not necessarily mirror the phylogenetic trend of the dinoflagellate lineages.

The distinct interspecific difference in gene copy organization is consistent to our observed clear *rbcII* sequence differentiation between species, as indicated in the monophyletic grouping of intraspecific *rbcII* gene copies (Figure 2). The monophyletic clustering of intraspecific gene copies is especially interesting in regard to the two *Prorocentrum* included in this analysis, *P. donghaiense* and *P. minimum*, because they are closely related [12] and share similar genome size and *rbcII* gene copy number. The species-specific *rbcII* sequence grouping suggests that the extensive duplication of this gene might have occurred within each species. Alternatively, it may be a result of strong sequence purifying selection imposed on each species [5]. In either case, this indicates a strong species-specific evolutionary process.

### Postulated signaling mechanism of PdRBC import

Protein trafficking pathways to plastids are directed by N-terminal targeting peptides [6]. Emerging through secondary endosymbiosis between photosynthetic and non-photosynthetic eukaryotes, typical dinoflagellate plastids are surrounded by three membranes [33]. Therefore, dinoflagellates must have developed a mechanism for nucleus-encoded plastid-targeted proteins to cross the three membranes and enter the plastid [9], [23], [33]. Nucleus-encoded, plastid-targeted protein genes in *L. polyedrum* contain an N-terminal leader sequence with two distinct hydrophobic regions flanking a region rich in hydroxylated amino acids (S/T) [23]. The same pattern also has been detected in *H. triquetra* Class I transit peptides [6]. The first hydrophobic region serves as a typical signal peptide, the S/T-rich region as a plastid transit peptide, and the second hydrophobic region as a stop transfer sequence in Golgi-derived vesicles [23]. The Golgi-derived vesicles are used for plastid protein shuttling between plastid membranes [33], [34], [35]. As in other dinoflagellate plastid-targeted proteins, PdRBC possess a bipartite N terminal targeting sequence formed by two hydrophobic peptides separated by a S/T rich chloroplast transit sequence. The bipartite targeting sequence has a signal peptide domain fused with a chloroplast transit peptide (cTP) [20], [36].

**Figure 6:**
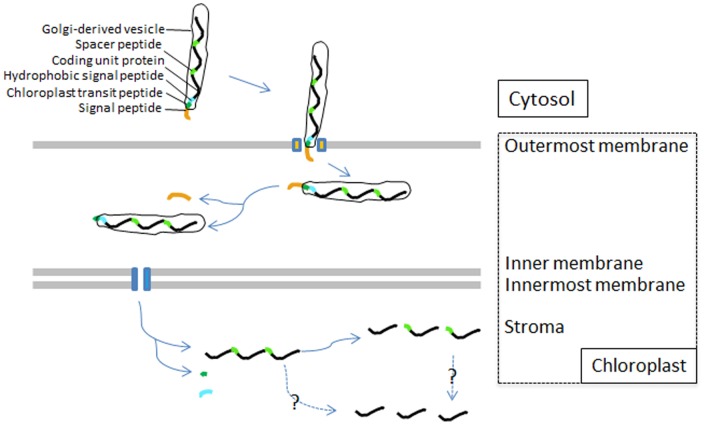
Schematic of PdRBC import pathway into the chloroplast in *P. donghaiense*. The precursor polypeptide consists of a signal peptide (orange) followed by a chloroplast transit peptide (deep green) and a hydrophobic signal peptide (blue). The signal peptide leads the polyprotein through the outermost membrane. The chloroplast transit peptide guides the polyprotein into the two inner membrane and then is cleaved off, unmasking the hydrophobic region peptide to stop transfer. The dashed arrows depict unknown cleave mechanism.

Since PdRBC N-terminus has a typical dinoflagellate plastid gene structure, it is reasonable to believe that PdRBC is imported into chloroplast in the same way as other reported dinoflagellate plastid-directed proteins. The presence of the peptide at the N-terminus of PdRBC suggests that the three CUs in each TU of *Pdrbc* must be co-translated and co-transported, as found in the 4-CU *P. minimum* RuBisCO [5]. The polyprotein PdRBC precursor should be led to endoplasmic reticulum (ER) membrane by its signal peptide and this allows the polyprotein's entry into the outmost membrane of chloroplast. (Figure 6; Figure S3). Presumably, the signal peptide is then cleaved. Next, guided by the cTP, the polyprotein is translocated to the two inner chloroplast membranes by vesicle-like cTP as in higher plant. Once in the destination, the polyprotein needs to be processed by removing the signal peptide to become single mature RBCII. When we analyzed the contiguous sequence that contains at least two tandem repeats and a spacer sequence, we identified a potential protease (3CFMDV) cleavage site (*) at WQKKE*LAAA, the juncture between the C-terminus of one CU and spacer sequence N-terminus of the next CU. Therefore, the cleavage would split the 3-CU PdRBC precursor to single-CU mature RBCII as reported in other dinoflagellates [4], [5]. Presumably, dinoflagellates possess a protease similar to FMDV polyproteins that form a cleavage mechanism to cut pre-mature RuBisCO, which however remains to be identified.

### The highly dynamic and cell cycle-dependent expression pattern of *Pdrbc*

It is believed that most genes in dinoflagellates are not transcriptionally regulated [9]. No transcriptional regulation of *RbcII* has been reported in dinoflagellates. In *L. polyedrum*, RBCII protein abundance was measured under different illumination conditions but no variation other than that in subcellular localization was observed [26], indicative of lack of transcriptional and translational regulations. It is thus striking to note the remarkable oscillation of RuBisCO transcript abundance in the light/dark cycle in *P. donghaiense*. When cells entered the dark period, *Pdrbc* transcript abundance was in a low level. Then it increased steadily from the middle of the dark period (h6) to reach a maximum at the dark-light transition (h10). During this period, G2M-phase cells also increased steadily to a peak level at h10 (Figure 3A). Whether previous failure to detect the transcriptional regulation of *rbcII* and other genes in other dinoflagellates is due to interspecific differences or to inadequate synchrony of the cell populations used in the experiments warrants further studies.

The coincidence of the dynamics of *Pdrbc* transcript abundance with the light/dark alternation and the variations of G2M-phase fraction suggests that the expression of *Pdrbc* may be modulated by the light dark cycle and/or associated with cell division. Alternatively, both *Pdrbc* expression and the cell cycle could be under circadian clock control. Light is a very important factor regulating the expression of form I RuBisCO gene (*rbcL*) in phytoplankton [37], [38]. The transcript abundance of *rbcL* usually reaches its maximum around the onset of the light period in cultured *Coccolithus pelagicus* and some natural phytoplankton populations [39], [40], [41], and then declines steadily in 12 h, before it increases again. These results are consistent to the apparent association of the *Pdrbc* expression peak with the dark/light transition we observed.

However, in *Synechococcus* spp. and other cynobacteria, the activation of *rbcL* transcription also appeared to be cell division related [39], [40]. It is conceivable that when cells are preparing to divide, division of the carboxysome, which contains many monomers of endogenous RuBisCO, requires increased expression of *rbcL* [42]. In eukaryotic algae, the RuBisCO-containing pyrenoid is functionally equivalent to carboxysomes in cyanobacteria [43] and presumably divides with cell division as well. This may explain why the expression of RuBisCO is apparently correlated with the G2M phase.

In order to distinguish the effect of LD cycle from that of cell cycle on *Pdrbc* expression, we shifted the previously synchronized cultures grown under 14∶10 light∶dark cycle to LL and DD, respectively. We found that under LL, while there was no light/dark cue, the cell cycle still showed a clear pattern of progression, and *Pdrbc* expression showed significant correlation with the G2M phase. It is thus evident that the *Pdrbc* expression is not directly regulated by light/dark conditions.

Whether a circadian control was responsible for the *Pdrbc* diel expression pattern observed for the LD and LL cultures can be determined from *Pdrbc* expression patterns in cultures shifted from LD to DD conditions. As rhythms under circadian clock control usually would persist at least 2–3 days after the LD to DD shift [44], [45], we would expect that the diel rhythm of *Pdrbc* would persist for at least one day after the LD-DD shift. In our case, the cultures shifted to DD showed strong cell cycle arrest in the G1 phase (>96.8% cells in G1 phase throughout the sampling period), and the expression level of *Pdrbc* showed no remarkable changes. The loss of the rhythm in *Pdrbc* expression within 24 h under DD condition rules out the possibility that *Pdrbc* expression and the cell cycle are both under a common circadian clock control.

Taken together, the results from our light/dark manipulation experiments as well as from literature indicate in concert that *Pdrbc* expression is regulated in a cell cycle-dependent fashion, in association with the G2M phase of the cell cycle. Whether *Pdrbc* expression and the cell cycle are regulated by some common mechanism, and whether such regulatory mechanism is evolutionarily entrained to (but not directly controlled by) the light/dark cues remain to be studied further.

## Conclusion

Dinoflagellate *P. donghaiense* possesses a typical dinoflagellate form II RuBisCO gene, which has duplicated extensively resulting in 117 copies distributed in 39 transcribed units as triple-CU tandem repeats. There is evidence that the tandem repeats in a transcribed unit are co-translated and co-transported, since the N-terminal plastid targeting sequence is located only in the first coding unit. In contrast to the lack of transcriptional regulation of *rbcII* and many other genes in dinoflagellates reported so far, the expression of *Pdrbc* is strongly regulated transcriptionally, in a cell cycle-dependent fashion.

## Supporting Information

Figure S1 **Diel growth pattern of** ***P. donghaiense*** **in the synchronized LD cultures.** A burst of cell division occurred between late dark period and early light period. Two bursts of cell division were noticeable, one from h4–6 and the other h10–14, resulting in 1.13 doubling d^−1^ or a specific growth rate of 0.76 d^−1^.
(TIF)

Figure S2 **Correlation between QTR-normalized** ***Pdrbc*** **mRNA abundance and percentage of G2M-phase cells (A) as well as time distance (B) from onset of the light period in the LD cultures.**
(TIF)

Figure S3 **Transmembrane helices of transit peptide in N-terminus of dinoflagellate form II RuBisCO from three species using web software Tmpred (** http://www.ch.embnet.org/software/TMPRED_form.html **).**
(TIF)
