# Supplementary figures and images for: Tandem Repeats, High Copy Number and Remarkable Diel Expression Rhythm of Form II RuBisCO in Prorocentrum donghaiense (Dinophyceae)
Source: PLoS One. 2013 Aug 19;8(8):e71232. doi: 10.1371/journal.pone.0071232 (PMC3747160; doi:10.1371/journal.pone.0071232)

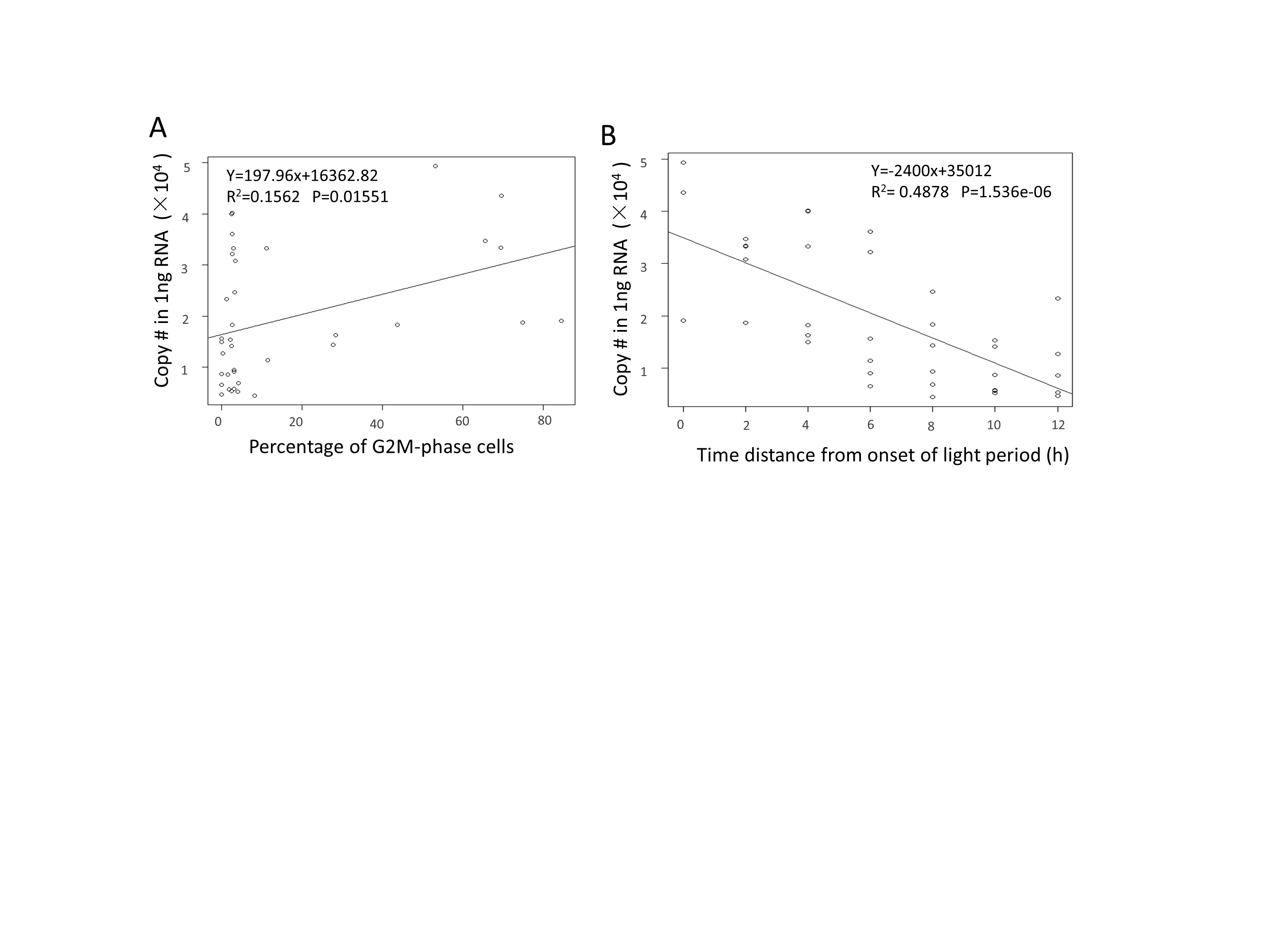

Supplement: Figure S2 — Correlation between QTR-normalized Pdrbc mRNA abundance and percentage of G2M-phase cells (A) as well as time distance (B) from onset of the light period in the LD cultures. (TIF) [file pone.0071232.s002.tif]

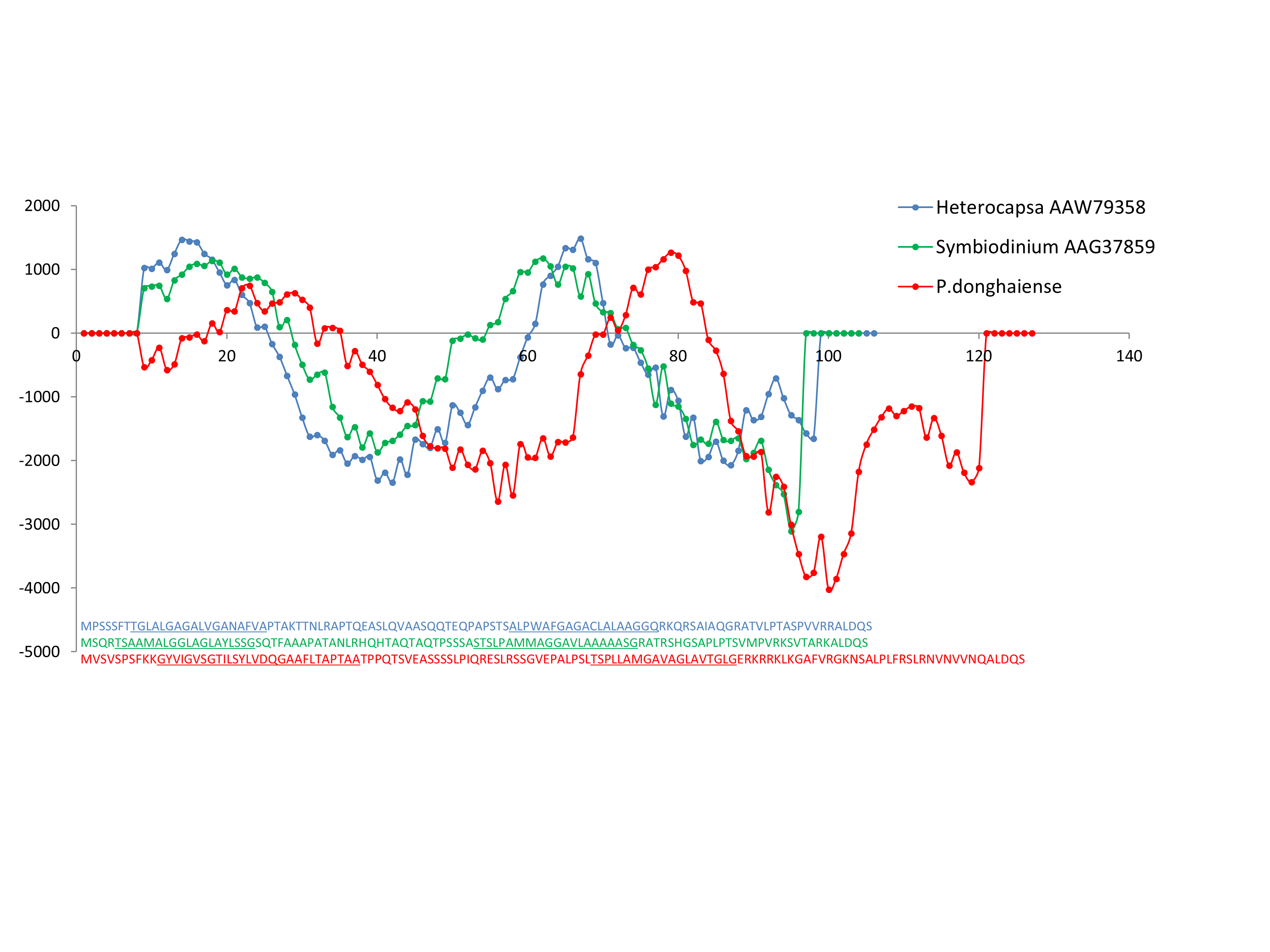

Supplement: Figure S3 — Transmembrane helices of transit peptide in N-terminus of dinoflagellate form II RuBisCO from three species using web software Tmpred ( http://www.ch.embnet.org/software/TMPRED_form.html ). (TIF) [file pone.0071232.s003.tif]
